# Complicated Hydatid Cyst: Ultrasonographic Illusion and Endoscopic Diagnosis

**DOI:** 10.5005/jp-journals-10018-1136

**Published:** 2015-01-06

**Authors:** Ayse Kefeli, Sebahat Basyigit, Abdullah Ozgur Yeniova, Yasar Nazligul

**Affiliations:** 1 Department of Gastroenterology, Siirt State Hospital, Siirt, Turkey; 2 Department of Gastroenterology, Kecioren Research and Training Hospital, Ankara, Turkey; 3 Department of Gastroenterology, Samsun State Hospital, Samsun, Turkey; 4 Department of Gastroenterology, Kecioren Egitim Ve Arastirma Hastanesi, Ankara, Turkey

**Keywords:** Echinococcus granulosus, Endoscopy, Ultrasonography.

## Abstract

Liver cyst hydatic (LCH) is an important parasitic disease caused by *Echinococcus spp.* Rupture of the biliary tract is an important and serious complication. It is often diagnosed on a suspicious clinical findings by radiologic imaging procedures. But, complicated hydatid cyst disease can show different radiological images, as they can simulate any disease. Here, we reported a case which diagnosed cholangiocellular carcinoma in primary diagnosis by ultrasonographic evaluation but diagnosed hydatid cyst in endoscopy evaluation. A 30-year-old female patient was admitted to the hospital with 1 year continuous severe abdominal pain aggravated for 1 month.

**How to cite this article:**

Kefeli A, Basyigit S, Yeniova AO, Nazligul Y. Complicated Hydatid Cyst: Ultrasonographic Illusion and Endoscopic Diagnosis. Euroasian J Hepato-Gastroenterol 2015;5(1):65-66.

To the Editor,

Liver cyst hydatic (LCH) is an important parasitic disease caused by *Echinococcus spp.* Rupture of the biliary tract is an important and serious complication. It is often diagnosed on a suspicious clinical findings by radiologic imaging procedures. But, complicated hydatid cyst disease can show different radiological images, as they can simulate any disease. Here, we reported a case which diagnosed cholangiocellular carcinoma in primary diagnosis by ultrasonographic evaluation but diagnosed hydatid cyst in endoscopy evaluation. A 30-year-old female patient was admitted to the hospital with 1 year continuous severe abdominal pain aggravated for 1 month. In physical examination, there is no positive clinical sign other than sensitivity of right abdominal quadrant. In her laboratory findings, it was found that 9500/ul of total leukocyte count, 11.7 gm/dl of hemoglobin, 270000/ul of platelet, 2.3 mg/dl of total bilirubin (T Bil), 1.5 mg/dl of direct bilirubin (D Bil), 668 IU/l of alkaline phosphatase (ALP), 207 IU/l of alanine aminotransferase (ALT), 357 IU/l of as partate aminotransferase (AST), 512 IU/l of gamma-glutamyl transferase (GGT). Abdominal ultrasonography showed a 10 × 9 cm size mass and a 93 × 36 mm size dense content cyst including solid component inside the mass which fills nearly whole right hepatic lobe. Dilatation of intrahepatic biliary tree was detected and common bile duct was 19 mm in diameter and a solid lesion inside the distal section of the lumen appeared like cholangiocellular carcinoma. Meanwhile, patients developed overt jaundice, total bilirubin increased to 10.8 mg/dl, body temperature was measured as 39 to 39.5°C. One gram of ceftriaxone was given twice daily. The patient underwent gastroscopy; a lesion colored yellow-white was thought to be hydatid cyst membrane inside Vater’s papilla extending into the duodenum (Fig. 1).

Endoscopic retrograde cholangiopancreatography (ERCP) was made due to increase in bilirubin level and endoscopic evidence of the hydatid cyst membrane. Amounts of cyst membrane and materials of cyst was removed by using biliary occlusion balloon. Biliary tract of right lobe cannot be visualized. Nasobiliary drain was placed in the right intrahepatic bile ducts. Agglutination tests for hydatid cyst was positive by 1/320 and 400 mg of albendazole was given twice a daily. By control ultrasound, it was observed to be an 85 × 75 mm hypoechoic echogenicity with dense content suggesting that cystic lesion, and echogenicity belongs to the air in the intra- and extrahepatic bile ducts in the right lobe. Laboratory findings showed 2.0 mg/dl of T Bil, 51 IU/l of ALT, 61 IU/l of AST, 420 IU/l of ALP. Abdominal pain and jaundice of the patient was decreased. She was discharged with a treatment of 400 mg of albendazole twice a daily. The patient had no symptoms. The ultrasono-graphical assessment showed that she was normal after 3 months.

Liver cyst hydatic is an important parasitic disease caused by *Echinococcus spp.* It is usually transmitted during childhood and remains silent lifelong. Jaundice with fever or even anaphylactic reactions may occur, if it is complicated. The most important complications of LCH are the infection of the cyst, obstructive jaundice due to compression or rupture of intrahepatic bile ducts, rupture through peritoneum, or the spread to other organs. Rupture of the biliary tract is an important and serious complication. It is often diagnosed on a suspicious clinical findings by radiologic imaging procedures.^1^

**Fig. 1:**
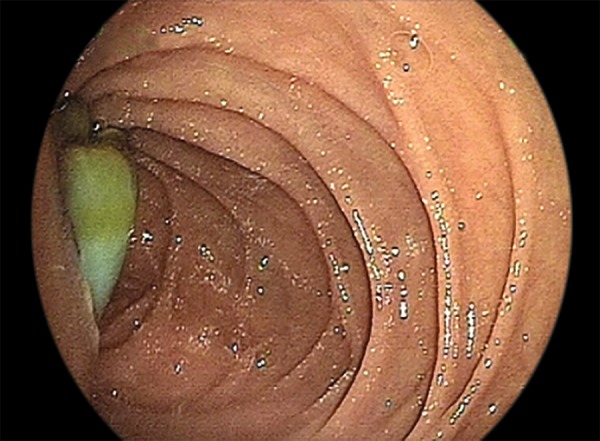
Hydatid cyst membrane protruding into the second part of duodenum from Vater’s papilla

Complicated hydatid cyst disease can show different radiological images, as they can simulate any disease. In our case, the sonographic examination of the 10 cm mass insisted to consider cholangiocellular carcinoma as a primary diagnosis. Endoscopic evaluation was made due to increased dyspepsia and overt jaundice on the 3rd day of admission and presence of yellow lesions extending into the duodenum.

It is uncommon to make diagnosis of intrabiliary rupture of LCH by regular endoscopic examination. Endoscopist should recognize the appearance of cyst membrane protruding into duodenum from Vater’s papilla and avoid removing it because of high inoculation risk. In this article, we reported the endoscopic appearance of cyst membrane of an intrabiliary rupture of LCH in the second part of the duodenum.
